# Liquid Silicone Rubber Headlamp Lens Injection Molding Process Optimization Based on Tie Bar Elongation and NSGA III

**DOI:** 10.3390/polym15214278

**Published:** 2023-10-31

**Authors:** Hanjui Chang, Shuzhou Lu, Yue Sun, Rui Wang

**Affiliations:** 1 Department of Mechanical Engineering, College of Engineering, Shantou University, Shantou 515063, China; 21szlu@stu.edu.cn (S.L.); 22ysun@stu.edu.cn (Y.S.); m18403985052@163.com (R.W.); 2 Intelligent Manufacturing Key Laboratory of Ministry of Education, Shantou University, Shantou 515063, China

**Keywords:** tie bar elongation, liquid optical silicone lenses, residual stresses, clamping force, optical transmittance

## Abstract

**Highlights:**

**What are the main findings?**

- The connection between cavity pressure, clamping force, and tie bar elongation is revealed and applied.
- The quality of the product is predicted based on the tie bar elongation, and the method is non-destructive, online, and highly efficient.

**What is the implication of the main finding?**

- The NSGA III algorithm can solve the multi-objective optimization problem while making the final obtained Pareto solution as well as being uniformly distributed.
- LSR lenses with low residual stress and high transmittance can be produced using this method.

**Abstract:**

This study aimed to improve the injection molding quality of LSR material lenses by optimizing the process parameters. To achieve this goal, we employed the population-based optimization algorithm NSGA-III, which can simultaneously optimize multiple objective functions and identify an equilibrium point among them, thereby reducing the time required to find the optimal process parameters. We utilized analysis software to simulate the injection molding process of LSR material lenses, with a specific focus on examining the relationship between tie bar elongation and the optimized process parameters. During the study, we intentionally varied key process parameters, including the melt temperature, holding pressure, and holding time, to analyze their impact on the residual stress of the final product. In order to investigate the intricate relationship between the tie bar yield, injection molding process parameters, and lens residual stress, we installed strain sensors on the tie bar to continuously monitor changes in clamping force throughout the injection molding process. The experimental results showed that both the tie bar force and mold cavity pressure exerted significant influence on residual stresses. By applying the NSGA-III algorithm for optimization, we successfully determined the optimal process parameters, which included a melt temperature of 34.92 °C, a holding pressure of 33.97 MPa, and a holding time of 9.96 s. In comparison to the initially recommended process parameters during the design phase, the optimized parameters led to reductions of 12.98% in clamping force and 47.14% in residual stress. Furthermore, the average transmittance of the actual product remained within the range of 95–98%. In summary, this approach not only enables the prediction of the lens’s residual stress trends based on the tie bar elongation, but also leads to a substantial enhancement of lens quality, characterized by reduced residual stress and improved transmittance through the optimization of process parameters. This methodology can serve as a valuable guide for optimizing real-world injection molding processes.

## 1. Introduction

Injection-molded lenses play a pivotal role as common optical components in a wide range of applications, including cameras, optical instruments, and automobiles. Nevertheless, the injection molding process is plagued by several challenges [1]. First, it is susceptible to molding defects such as bubbles, fusion lines, and shrinkage, all of which can profoundly impact product quality and performance and even lead to outright failure. Second, optical defects such as aberration, astigmatism, and chromatic aberration can arise within the injection mold, diminishing the practical utility of the final product. Lastly, the dimensional accuracy of injection molds is frequently compromised and influenced by factors such as temperature, pressure, and mold precision [2].

Researchers have harnessed sensing technology to enhance injection molding production by optimizing processes and monitoring quality [3,4,5]. Through the strategic installation of sensors to oversee variations in clamping force, injection pressure, and mold cavity pressure, the injection molding process is stabilized, ensuring consistent and reliable product quality. The invaluable feedback furnished by these sensors empowers operators to make real-time adjustments to parameters, thus guaranteeing effective plastic filling of molds and the maintenance of uniform product quality. The deployment of pressure sensors in the injection molding process serves as a critical pillar in process control and quality assurance.

In 2022, Chang et al. [6] presented a real-time product-quality-monitoring system based on clamping force variations for injection-molded caps made from recycled plastics. The clamping force variation correlates with cavity pressure fluctuations during the injection process, and this relationship is intertwined with injection parameters, product quality, and molding conditions. This approach has proven instrumental in monitoring product quality, enhancing production capacity, and curbing costs. In 2019, Huang et al. [7] introduced a real-time quality-monitoring system applicable to both raw materials and recycled plastics. This system relies on incremental clamping force measurements and the precise control of the finished product weight through V/P switching point calibration. In 2018, Zhao Peng et al. [8] proposed a method to measure cavity pressure using ultrasonic technology calibrated using a magnetic clamping force detector. The method is accurate, reliable, and suitable for the online monitoring of injection molding processes and product performance evaluation, and it has great potential for popularization and application.

Mold cavity pressure directly affects product quality and performance, and it is critical to control cavity pressure [9,10]. Pressure that is too high or too low can lead to defects such as warpage, underfill, and bubbles. Adjusting the injection parameters, optimizing the mold design, and selecting the right material can control cavity pressure and ensure product quality [11]. Process parameters have a direct impact on product quality, productivity, and cost, and it is critical to understand and control them accurately. However, injection molding processes are complex, and traditional manual adjustments make it difficult to achieve optimal results [12]. Therefore, sensor monitoring and control of the injection molding process combined with the proper adjustment of process parameters is key.

In 2015, Jian Zhao et al. [13] used a global optimization algorithm to nonlinearly fit the process parameters and optimization objectives and used NSGA II to optimize the parameters of the three optimization objectives of dent displacement, warpage, and volume shrinkage, and their results showed that the Pareto obtained by the NSGA-II algorithm was convergent and robust. In 2015, Zhang et al. [14] used artificial intelligence to optimize the cooling channel design of an oil cooler cover. The optimized product showed reduced warpage and improved stress distribution. In 2019, Alejandro Alvarado-Iniesta et al. [15] proposed an optimization model with up to seven objectives using plastic gears as a research object and a Pareto Explorer for the global or local exploration of multiple objectives. The proposed approach can provide more alternatives and guidance for decision makers. In 2022, Chang et al. [16] proposed a combined nodal optimization and genetic algorithm for the optimal design of UAV shells. The method effectively reduced product warpage and optimized mold indices, with the predicted results aligning well with actual production. 

**Figure 1:**
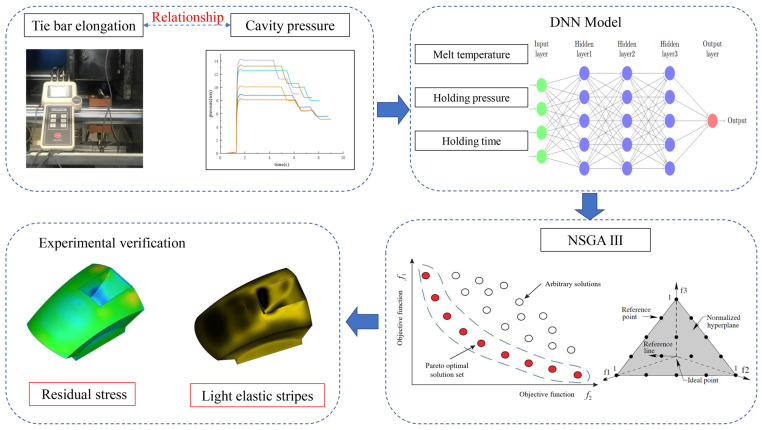
NSGAIII Optimization Concept for optical components experimental.

As evident from the aforementioned studies, the amalgamation of artificial intelligence algorithms proves highly effective in analyzing and optimizing injection molding process parameters [17,18,19]. By aggregating and scrutinizing extensive historical data, sensor data, and product quality data, AI algorithms can construct predictive models and correlation models that forecast the effects of varying process parameters on product quality. This enables the identification of the optimal combination of process parameters. [20] In line with the focus of this study, which centers on the interplay between tie bar elongation, cavity pressure, and lens quality, a DNN was utilized to establish a mathematical model linking these three process parameters to the objective function. Subsequently, the NSGA-III algorithm was employed for the multi-objective optimization of parameter settings, leading to the identification of the best process parameters via the Pareto front. Finally, transmissivity testing was conducted to validate the feasibility of the experimental method, as illustrated in Figure 1.

## 2. Material and Methodology

### 2.1. Liquid Silicone Rubber

Liquid silicone rubber (LSR) is a high-purity, low-viscosity liquid silicone rubber material with many excellent properties, such as high-temperature stability, chemical resistance, electrical insulation, and low-temperature flexibility. It is a polymer material made of silicone-based monomers such as dimethyl siloxane (DMS) and dimethyl siloxane polymerized under the action of an acid catalyst (DMSK). LSR is usually used to manufacture high-precision, high-quality rubber products. Due to its low viscosity and good fluidity, it is possible to manufacture combined shape parts with high precision and surface finish by injection molding and other methods. LSR also has good anti-aging properties and can be used to manufacture products for long-term use, such as automotive parts, medical devices, and electronic products. However, the manufacturing process of liquid silicone rubber requires the strict control of parameters such as temperature, pressure, and time to ensure product quality and performance.

Table 1 provides a comprehensive comparison of three different materials: LSR, polycarbonate (PC), and polymethylmethacrylate (PMMA). It covers a variety of key properties, including chemical composition, physical state, heat resistance, elasticity, chemical resistance, electrical insulation, transparency, processing methods, and typical uses. This comparative table helps engineers, designers, and manufacturers better understand the properties of these materials to make informed material choices for a variety of applications.

**Figure 2:**
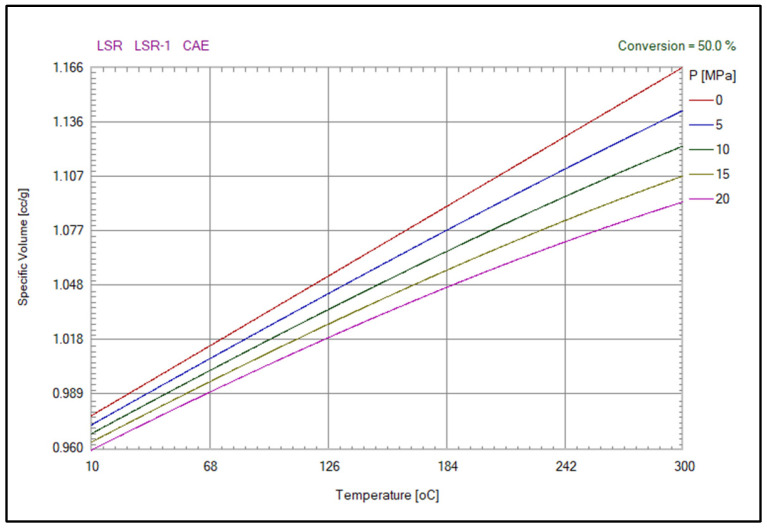
PVT curves of liquid silicone materials used for lenses.

**Figure 3:**
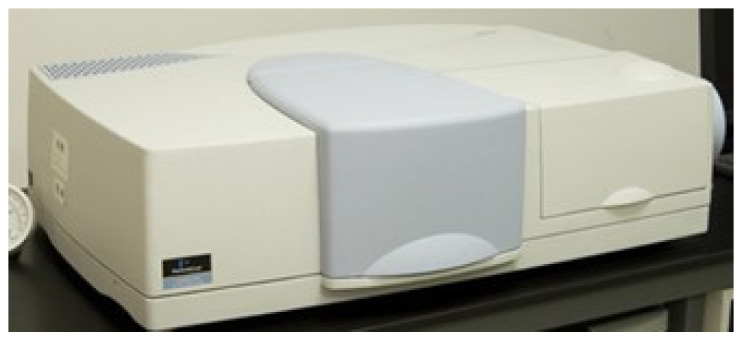
UV–visible near-infrared spectrophotometer.

The PVT curves of LSR in Moldex-3D’s material database are shown in Figure 2. The curves show the relationship at different pressures (0–20 MPa), volumes, and temperatures. By analyzing the PVT curve, the physical and chemical properties of liquid silicone rubber under different conditions can be determined, and then its processing process and application characteristics can be optimized. The PVT curve is also one of the important indicators for assessing the thermodynamic stability and phase change characteristics of liquid silicone rubber. In addition, the feasibility of this method was verified in this study by performing a quality check of the transmittance of the finished LSR lenses using a Lambda 950 UV/VIS spectrometer manufactured by PerkinElmer, Waltham, MA, USA (Figure 3).

### 2.2. Optimization Method

To find the optimal injection molding process parameters and their corresponding best response values quickly and reduce the manual debugging cost, we mainly applied NSGA-III (non-dominated sorting genetic algorithm III), which is a theoretical algorithm for the optimization of engineering problems modeled after biological evolutionary mechanisms, to optimize process parameters. It was applied to find optimal solutions or solution sets from new populations using an iterative approach. It is an extended version of NSGA-II based on the concept of non-dominated ranking and is used to classify populations into different frontiers according to their fitness values.

NSGA-III uses the reference point method to generate solutions that are uniformly distributed over the Pareto frontier. The reference point approach involves setting a reference point for each target that is used to guide the search toward the Pareto frontier. The algorithm then generates a set of solutions that are as close as possible to the reference point while ensuring that they are non-dominated.

NSGA-III is particularly suitable for solving problems with a large number of targets because it can handle a large number of targets without becoming computationally expensive. It is also able to generate a diverse set of solutions evenly distributed over the Pareto front, which makes it useful for decision making in situations where multiple conflicting objectives need to be considered.

**Figure 4:**
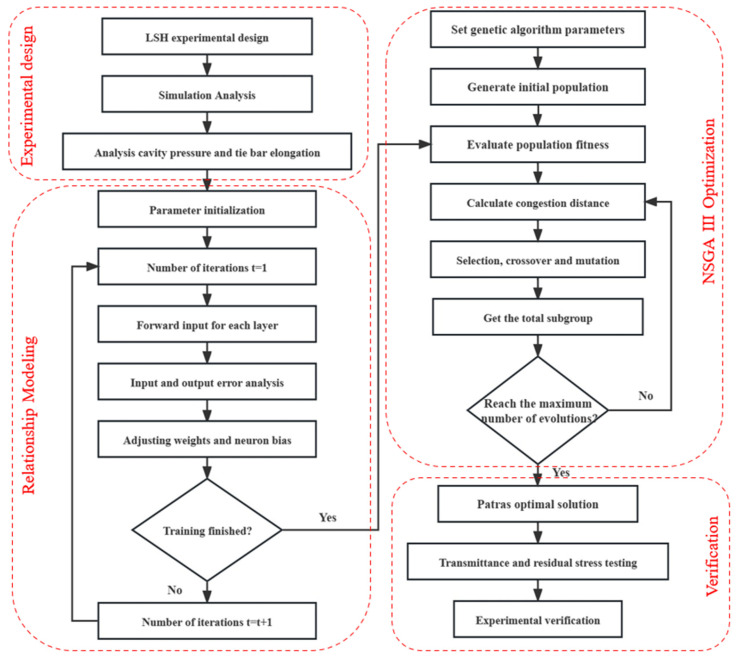
Flow chart of the genetic algorithm.

NSGA-III is a multi-objective optimization algorithm that can be used to optimize lens injection molding. Figure 4 shows the optimization steps of lens injection molding based on NSGA-III: (1) The optimization target is determined, with the residual stress and clamping force of the lens as the optimization target. (2) The optimization model of lens injection molding is established, setting parameters such as injection temperature, pressure, speed, and the cooling system. (3) According to the optimization target and optimization model, multi-objective optimization is carried out to optimize the injection parameters. Through the NSGA-III algorithm, a set of Pareto optimal solutions can be obtained, where each solution is a set of optimal injection molding parameters. (4) Experimental validation is conducted to verify the feasibility and stability of the optimization results. The experimental process requires attention to data collection and analysis for further optimization and improvement.

The fast non-dominated sorting algorithm of NSGA-III is as follows:

Suppose there are m objective functions. S is the set of individuals to be sorted, N denotes the size of the set S, R denotes the rank of the sort, F(i) denotes the function value of individual i on the rth objective function, D(i,j) denotes the distance between individual i and individual j, and *C*(*i*) denotes the number of individuals that dominate i.

In the first step, the counter C(i)=0 is initialized to classify all individuals as the first rank, i.e., no individual dominates that individual.

In the second step, the distance value D(i,j) is calculated for each individual i and sorted according to the distance value from smallest to largest.

In the third step, the preceding individuals are classified as the first rank and the following individuals are classified as the second rank until all individuals are assigned to the rank.

In the fourth step, the dominance relationship is judged based on the current ranking level r and the individuals in the previous ranking level r−1. If individual i dominates individual j, then 1 is added to the counter of C(j). Then, the value of C(i) is set to the number of j.

In the fifth step, if the number of individuals in the current rank exceeds the total number N to be selected, then the first N individuals are selected based on the distance value of each individual; otherwise, the process is repeated in the next rank.

The important formulas of NSGA-III are as follows:

1. Equation (1) for calculating congestion:
  (1) $$C(i)=\sum_{j=1}^{M}\frac{F_{j}(i+1)-F_{j}(i-1)}{f_{j,max}-f_{j,min}}$$
2. Equation (2) for calculating the distance:

(2) $$D_{i,j}=\sum_{k=1}^{M}\frac{(F_{k}(i)-F_{k}(j){)}^{2}}{f_{k,max}-f_{k,min}}$$

where $C\left(i\right)$ is denoted as the crowding degree, $D_{i,j}$ is denoted as the distance from individual *i* to individual j, *M* denotes the number of objective functions, $F_{j}(i)$ denotes the function value of individual *i* on the jth objective function, and $f_{j,max}$ and $f_{j,min}$ denote the maximum and minimum values of the jth objective function among all individuals, respectively.

**Figure 5:**
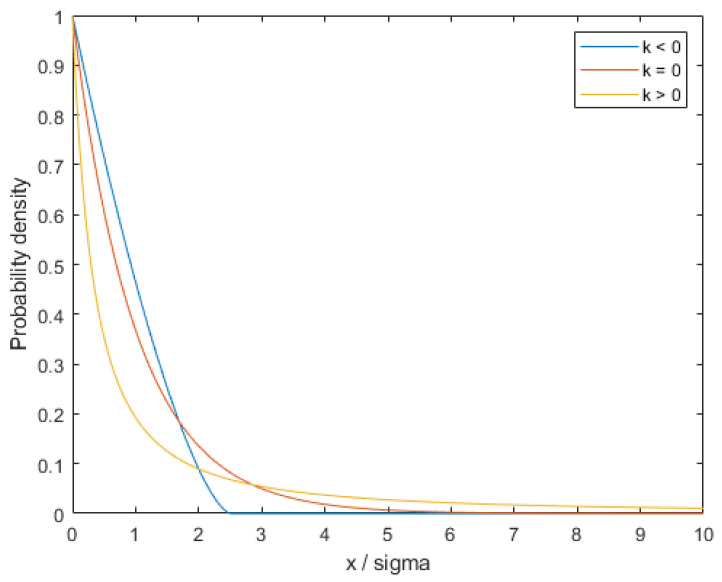
Pareto distribution for three different shape parameters k.

As shown in Figure 5, the Pareto distribution, also known as the Zipf distribution, is a continuous probability distribution in probability theory and statistics. It is usually used to describe some random phenomena with a long-tail nature, such as wealth distribution, city population distribution, and number of web links. The probability density function of this distribution is relatively simple in form but has a heavy-tailed nature, which means that it decays very slowly in the tail. It is parameterized using the shape parameter k and the scale parameter sigma. k is also known as the “tail index” parameter and can be positive, zero, or negative.

In injection molding processing, it is common to encounter the challenge of controlling the dimensions of production parts. Factors such as material variations, process fluctuations, and equipment wear and tear, among others, can lead to deviations in part dimensions. To maintain product quality, it becomes essential to inspect and control these part dimensions. In this context, the Pareto distribution can be effectively employed to characterize the distribution of part dimensions.

In injection molding, part dimensions usually obey the Pareto distribution. In other words, many part sizes are distributed in a small range, but a few parts will have large sizes. These larger sizes are called outliers and are usually caused by chance in production. Due to the heavy-tailed nature of the Pareto distribution, the presence of these outliers can have a large impact on the shape of the overall distribution.

Therefore, to control the quality of the part dimensions, some measures need to be used to limit the appearance of outliers. For example, some process improvement measures, such as adjusting the pressure and temperature parameters of the injection molding machine, can additionally be used to reduce the appearance of dimensional deviations. In conclusion, the Pareto distribution can be used in the injection molding process to describe the distribution of part dimensions and also help to develop reasonable production control measures to improve product quality.

### 2.3. Experiment

**Figure 6:**
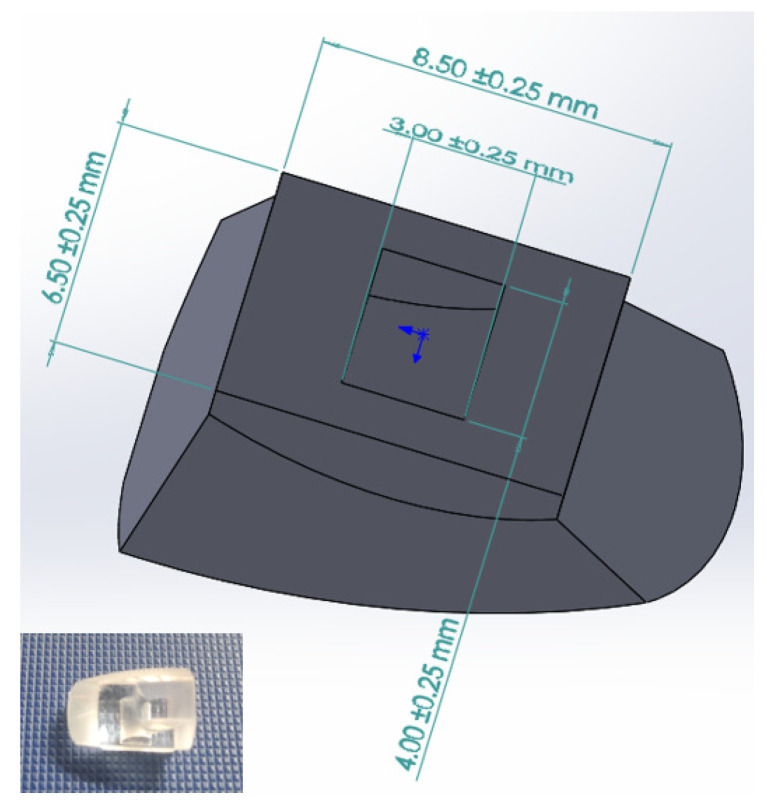
Photo and three-dimensional drawing of an optical silicone lens.

In this study, a liquid optical silicone component for automobiles produced by a specific company was selected for the study. This component is made by LSR material injection molding and has a complex internal structure and special dimensional requirements. To study the structural characteristics and performance of this component, we conducted dimensional measurements and modeling analysis. The measurement results revealed that the liquid optical silicone component has dimensions of 13.88 mm×9.88 mm×6.52 mm, with a maximum thickness of approximately 10.37 mm, a minimum thickness of about 0.057 mm, and a total volume of $384.97\;{\mathrm{mm}}^{3}$. During the modeling process, we used SolidWorks 2020 software and combined the functions of stretching, rotation, and array to make the established model show the structural characteristics of the component comprehensively. Ultimately, we successfully developed a 3D model of the liquid optical silicone component for the vehicle, as depicted in Figure 6.

During the injection molding process, tie bars are used to clamp the mold and to measure the clamping force using strain sensors. The clamping force is the pressure between the fixed and moving mold, reflecting how tightly the mold is clamped. The pressure in the mold directly affects the quality of the molded part. By measuring the clamping force with strain sensors, we can infer the pressure in the mold. During the injection molding process, the molten plastic in the mold generates a certain amount of pressure. The pressure in the mold cavity directly affects important parameters such as the size, density, surface quality, and mechanical properties of the plastic part, so it must be controlled within a certain range. If the pressure in the mold cavity is too high or too low, then it may lead to product quality problems. For example, if the pressure is too high, then it may lead to excessive shrinkage of the part, surface defects, large dimensional deviations, and other problems; if the pressure is too low, then it may lead to insufficient density of the product, degradation of mechanical properties, and other problems.

Injection molding machine tie bars are used to clamp the mold, and strain sensors on the tie rods are used to measure the magnitude of the clamping force in real time. A strain sensor on the tie rod can also be used to measure the clamping force. The principle is that the sensor converts the pressure into an electrical signal related to the strain change during operation, thus directly measuring the clamping force and enabling the real-time monitoring and evaluation of the tie rod strain state. The stress of each tie rod is calculated using Equations (3) and (4) and the clamping force is determined using Equation (5).

(3) $$\varepsilon_{i}=\frac{F_{i}}{EA}$$

(4) $$F_{i}=\frac{EA\varepsilon_{i}\times 10^{9}}{9.81}$$

(5) $$F=\sum_{i=1}^{n}F_{i}$$

where $\varepsilon_{i}$ is the stress of the *i*th tie in microns, *E* is Young’s modulus of the tie (=210,000 kgf/cm^2^), *A* is the cross-sectional area of a single tie in square millimeters, $F_{i}$ and *F* represent the ith tie and the total clamping force in tons, respectively, and *n* is the number of ties.

**Figure 7:**
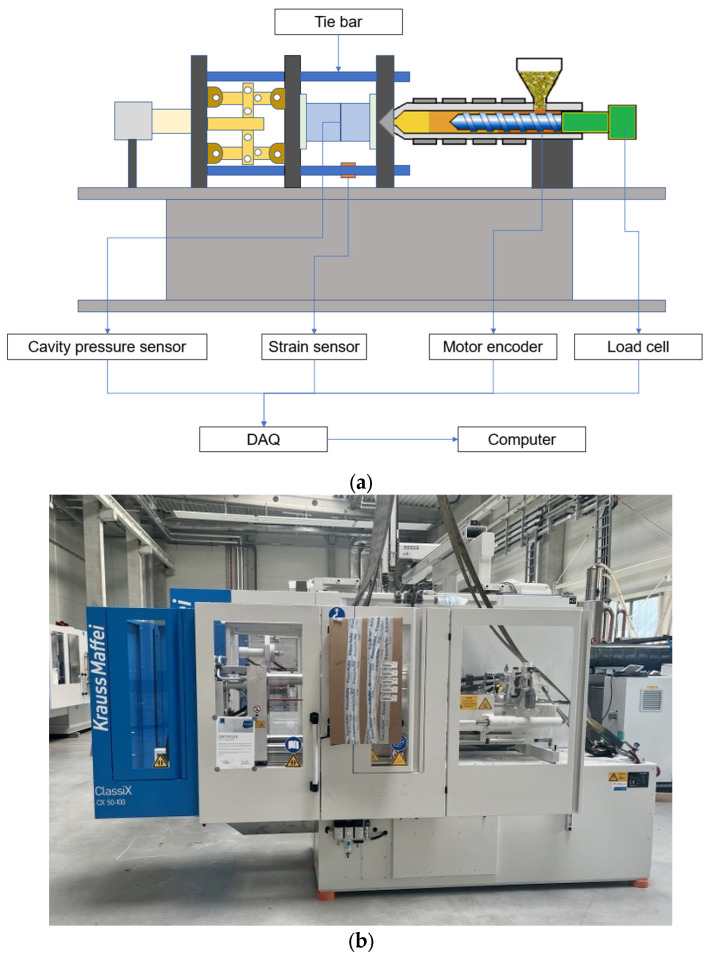
Experimental setup: (**a**) schematic diagram of the setup; (**b**) injection molding machine model KM 50–100 CX.

In addition, the structure of the experimental measurement of lens injection molding in this study is schematically shown in Figure 7, including an injection molding machine, strain sensors, a data acquisition (DAQ) interface, and a computer. Among them, four strain sensors are mounted on each of the four tie bars of the injection molding machine. The injection molding machine accurately measures the clamping force acting on the mold halves during the injection process through the tie bar strain sensors to achieve online detection of tie bar elongation changes. Therefore, installing strain sensors to measure the clamping force during the injection molding process and inferring the pressure inside the mold cavity based on the clamping force can help better control the parameters of the injection molding process to ensure the production of high-quality plastic parts.

## 3. Results and Discussion

**Figure 8:**
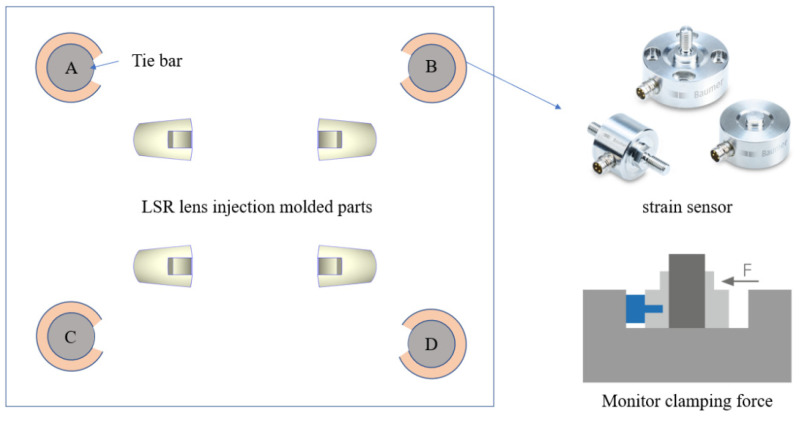
Schematic diagram of tie bar elongation test for injection molding machines. (A, B, C and D are the four tie bars of the injection molding machine).

As depicted in Figure 8, this study assessed variations in clamping force within the injection molding machine, serving as a quality indicator for the injection-molded product. This assessment was based on the analysis of strain changes along the surfaces of the tie bars, monitored by strain sensors affixed to all four tie bars of the injection molding machine. These strain measurements indirectly capture alterations in the internal pressure of the mold cavity, thus determining the product’s quality. In essence, the fluctuation of the strain sensor on the tie bar serves as a key indicator of the lens product’s quality.

The injection pressure and curing pressure were raised from 20 to 60 MPa in a series of experiments and the results are presented in Table 2. Based on the data in Table 2, it becomes evident that there is a correlation between the clamping force and cavity pressure of the injection molding machine. Furthermore, the strain data on the tie bar serve as an indicator of the fluctuations in the clamping force of the injection molding machine throughout its operation. The connection among these data can be elucidated in the following ways: First, the clamping force of the injection molding machine corresponds to the mold’s closing force during the injection process. This force results from the combined influence of cavity pressure and other factors. Hence, cavity pressure and clamping force can be regarded as two interrelated variables. As evident from the table, an increase in cavity pressure leads to a concurrent increase in clamping force. This implies that the cavity pressure directly impacts the clamping force of the injection molding machine. 

Second, the strain data on the tie bar mirrors the variations in clamping force during the operation of the injection molding machine. According to the data in the table, when the clamping force of the injection molding machine rises, the strain data on the tie bar also increase. This signifies that the strain data on the tie bar can serve as an indicator to represent the clamping force of the injection molding machine.

Lastly, the alteration in residual stress within the injection-molded component likewise mirrors the shift in pressure within the mold cavity during the injection molding process. When the pressure inside the mold cavity rises, the residual stress in the injection-molded part decreases correspondingly. Consequently, the residual stress in injection-molded parts can be employed as an additional indicator to depict changes in pressure within the mold cavity.

In summary, cavity pressure, clamping force, tie bar strain, and residual stress in injection-molded parts represent interconnected variables within the injection molding process. Through their monitoring and analysis, we gain insights into the fluctuations in the mold cavity and the operational status of the injection molding machine. This understanding enables us to optimize the injection molding process, ultimately enhancing both the quality and productivity of the injection-molded products.

**Figure 9:**
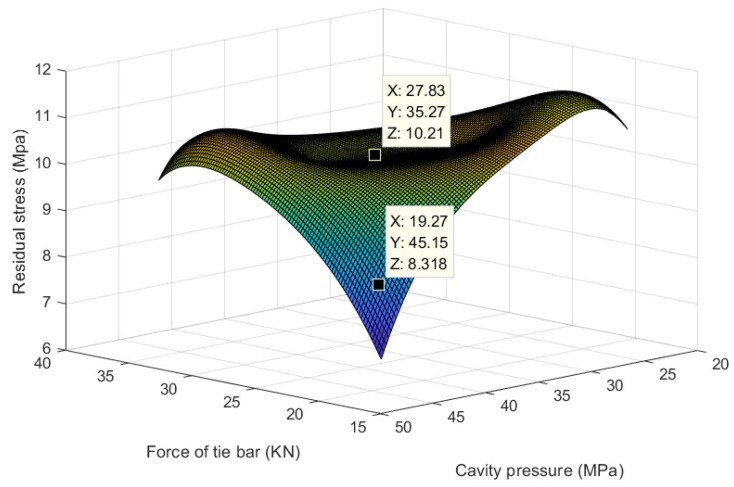
Diagram of the relationship between cavity pressure, the force of the tie bar, and residual stress.

Based on Figure 9, it becomes evident that the residual stress values do not exhibit a monotonic increase or decrease across the range of tie bar force and cavity pressure variables. In fact, they display distinct trends for different combinations of tie bar force and cavity pressure. As observed in the data plot, residual stresses tend to be lower in the central region and higher on both ends within the spectrum of tie bar force and cavity pressure. This implies that the residual stress values are elevated when the tie bar force and cavity pressure are either small or large, whereas within the intermediate range, the residual stress values are comparatively lower. This pattern may be attributed to the fixed mold force and mold cavity pressure, providing further evidence of the significant influence of tie bar force and cavity pressure on residual stress during the injection molding process.

It should be noted that we have provided a limited number of data points, and as such, we may not be able to comprehensively illustrate the impact of the tie bar force and cavity pressure on residual stresses. However, these data points allow us to gain an initial insight into the correlation among these three variables and offer a reference for optimizing the injection molding process.

In order to identify the critical control parameters influencing the optical properties of the lenses, we selected three process parameters that are closely linked to lens quality based on the literature review. These parameters include melt temperature (A), curing pressure (B), and curing time (C) serving as the test factors, while the optimization objectives encompass the clamping force and residual stress values of the silicone lens arrays. The recommended range of values for the levels of the test factors is shown in Table 3, and quantitative analysis experiments were conducted for these three main process parameters.

Based on the Latin hypercube sampling method for these three test factors, we generated 20 sets of Latin test samples, aligning with the test factor levels outlined in Table 3. The optimization objective results were obtained using Moldex3D Studio 2023 software as shown in Table 4, including the mean values of the residual stress and clamping force in columns 4 and 5, respectively.

In this paper, we leveraged MATLAB’s Machine Learning Toolbox and Optimization Toolbox to establish the necessary infrastructure for training neural networks and conducting optimization. As the input variables, we considered three crucial process parameters: melt temperature, holding pressure, and holding time, while utilizing residual stress and clamping force as the outputs variables. These were employed in the development of a deep neural network (DNN) for both training and testing. A DNN, characterized by its capacity to process extensive datasets, proves particularly apt for addressing the complexities of nonlinear problems in our study.

Following the construction of the neural network, we optimized the process parameters using the non-dominated ranking genetic algorithm (NSGA-III), aiming to minimize the residual stresses and clamping forces. NSGA-III is a multi-objective optimization algorithm capable of simultaneously optimizing multiple objective functions, which can sometimes lead to conflicts between different objectives.

Ultimately, we employed MATLAB’s drawing tool to visualize the Pareto boundary by plotting the optimization outcomes derived from the NSGA-III algorithm. This boundary encompasses the set of all non-dominated solutions, signifying that no alternative solution surpasses it across the various objective functions. The Pareto boundary serves as a valuable aid in identifying the optimal solution, thus facilitating the achievement of the goal of minimizing residual stresses and clamping forces.

**Figure 10:**
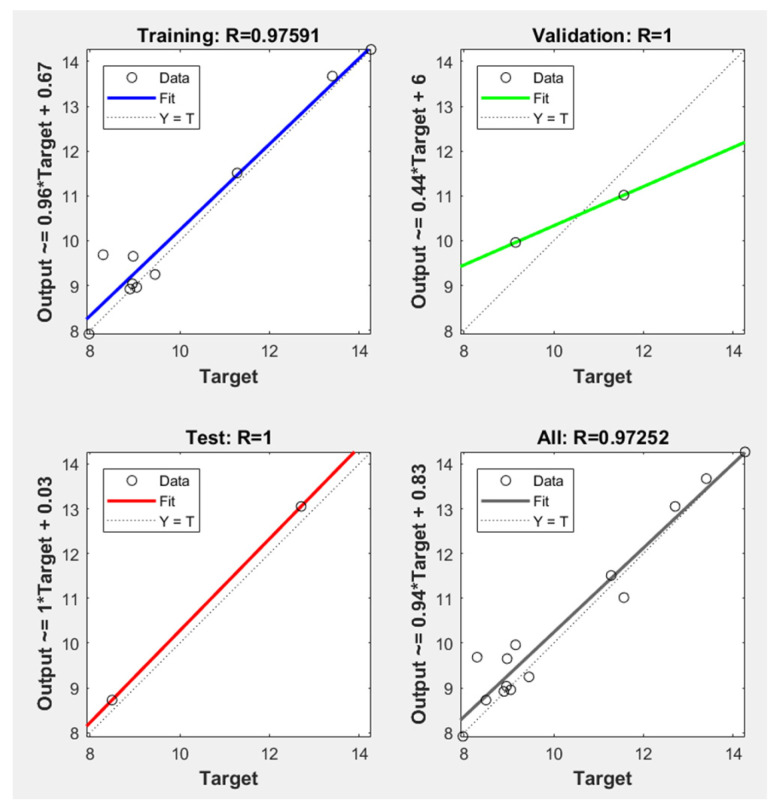
Performance of DNN models on the test set.

These factors exhibit varying magnitudes, and directional anisotropy was selected as the basis for constructing the model. A DNN model was constructed for prediction and the R2 metric was used to measure the performance of the model. Figure 10 presents the outcomes obtained through the application of the DNN for quality prediction. As depicted in the regression model plot, it is evident that the prediction results for the training set are highly accurate, underscoring the precision and quality of the prediction model. Furthermore, we conducted 10 cross-validation tests, and our DNN model consistently demonstrated strong performance in predicting the quality of injection-molded parts. In each case, the R2 value exceeded 0.9, signifying the significance of this surrogate model. Figure 10 illustrates the relationship between actual and predicted values of sample points, where data points are symmetrically distributed around the ideal straight line. This distribution confirms the model’s high accuracy and its suitability for fitting response values under varying factor combinations.

**Figure 11:**
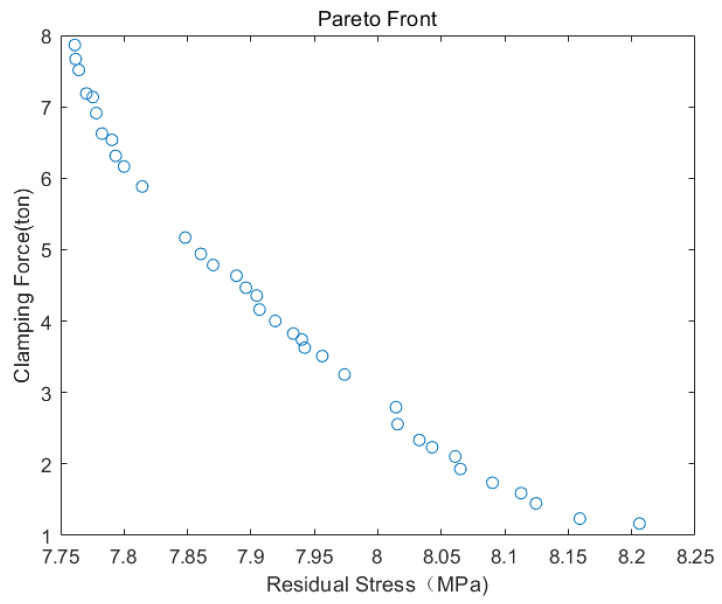
The Pareto frontier with double optimization objectives.

The set of Pareto optimal solutions is illustrated in Figure 11. Analysis of the Pareto optimal solution set reveals a mutual constraint between the two optimization objectives. It becomes evident that there is no combination of process parameters that simultaneously optimizes both objectives. Therefore, selecting the most suitable set of solutions is necessary according to different weights. Following global optimization using NSGA-III, the optimal process parameters are determined as follows: 34.92 °C for melt temperature, 33.97 MPa for curing pressure, and 9.96 s for curing time. As shown in Table 5, the residual stress and clamping force were reduced by 12.98% and 47.14%, respectively, compared to the initially recommended process parameters. This signifies a significant enhancement in optimization outcomes. 

**Figure 12:**
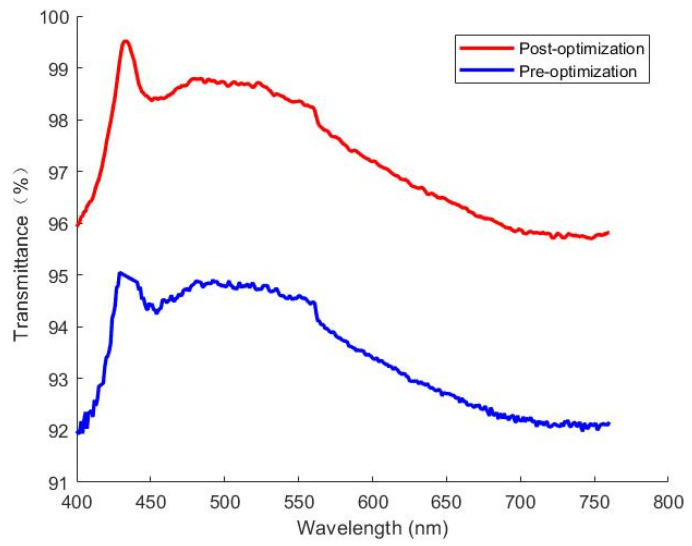
LSR lens transmittance test results in the visible light range (blue line is the residual stress of 14.271 MPa before optimization, red line is the residual stress of 7.544 MPa after optimization).

The process parameters were designed according to the results of the mode flow analysis, the optimized process parameters were used in the trial mold production to produce the LSR lens products, and the transmittance test results of the actual products are shown in Figure 12. Under the same situation, the quality of the low residual stress lens is better, and its transmittance can be kept above 96%, while the transmittance of the high residual stress lens can only reach 92–96%; therefore, the low residual stress lens used in the light path system and lighting system will obtain higher light energy utilization and uniform lighting distribution over a large area.

In the existing research the main measurement of mold cavity pressure is the pressure sensor method, but the pressure sensor method is detrimental to the mold, and it is difficult to achieve industrial large-scale promotion, so we propose using the strain gauge method for online measurement of the force on the tie bar of the injection molding machine, and then realizing the measurement of the clamping force of the injection molding machine and the mold cavity pressure, which is of great significance for improving the working life of the injection molding machine, enhancing the quality of the injection-molded products, and reducing the production cost.

## 4. Conclusions

This paper presents a new method for measuring tie bar stresses in injection molding machines using strain sensors, which enables the simultaneous measurement of clamping force and cavity pressure. The method is online, non-destructive, cost-effective and suitable for large-scale application. It effectively addresses the challenge of precise online measurement of tie bar stress, clamping force, and mold cavity pressure in industrial production process. Moreover, when combined with the NSGA-III artificial intelligence algorithm and systematic adjustment of process parameters, it can reduce the residual stress in the lens and improve the optical penetration of the product.

1. A mathematical method for measuring large column strains to estimate mold cavity pressure is outlined. Real-time monitoring and analysis of these parameters not only offer insight into the internal pressure of the mold cavity, but also unveil the dynamic operating conditions of the injection molding machine.
2. The core focus of this part is the optimization of process parameters for injection molding lenses, achieved through the use of a deep neural network (DNN) and a non-dominated sorting genetic algorithm (NSGA-III). Importantly, Pareto boundaries play a crucial role in identifying the most advantageous solution, one that effectively minimizes both residual stresses and clamping forces.
3. After global optimization through NSGA-III, the study successfully identified the best process parameters. These optimal settings included a melt temperature of 34.92 °C, a curing pressure of 33.97 MPa and a curing time of 9.96 s. It is noteworthy that the residual stress and clamping force were reduced by 12.98% and 47.14%, respectively, compared to the process parameters initially recommended at the design stage. This outcome underscores the remarkable effectiveness of the optimization process, leading to significant improvements.
4. In addition, a correlation between tie bar elongation, mold cavity pressure, and lens quality was observed. It was noted that as the tie bar elongation increased, there was a corresponding rise in the pressure within the mold cavity. This increase in pressure, in turn, led to a reduction in residual stresses within the product after demolding. In practical applications, such as trial mold production, engineers can utilize the monitored tie bar elongation to fine-tune process parameters, ultimately achieving a minimization of residual stress within the product.

In the study of installing sensors on the pull rod of injection molding machines to detect pull rod stress and subsequently infer clamping force and product quality, there exist extensive practical applications and avenues for future research. First, our research provides valuable tools for the manufacturing industry, enabling automated quality control, real-time monitoring of product quality, reduction in defect rates, enhanced production efficiency, and lowered production costs. Furthermore, seamlessly integrating sensor data into automated production lines facilitates system integration, rendering the production process more intelligent and efficient.

The potential for future research encompasses the development of more sophisticated data analysis and machine learning models for quality prediction and early detection of potential issues. Additionally, exploring multi-sensor applications holds promise for further optimizing material selection and process parameters, thereby advancing sustainable manufacturing. These practical applications and future research directions inject fresh vitality into the field of injection molding, fostering continuous progress and improvement. Hence, this study establishes a robust foundation for quality control in the injection molding process, encouraging further in-depth exploration in the future.

## Figures and Tables

**Table 1:** Comparison of the properties of LSR, PC, and PMMA materials.

Property	LSR	PC	PMMA
Physical State	Liquid	Solid	Solid
Heat Resistance	High	High	Moderate
Elasticity	Excellent	Good	Good
Chemical Resistance	Excellent	Good	Fair
Electrical Insulation	Excellent	Good	Excellent
Transparency	High Transparency	Transparent or Translucent	High Transparency

**Table 2:** Experimental measurement of cavity pressure, clamping force, tie bar stress, and residual stress.

Items	Cavity Pressure (MPa)	Clamping Force (ton)	Force of Tie Bar (KN)	Residual Stress (MPa)
1	1.799	0.594	1.485	12.698
2	23.280	7.137	17.843	10.752
3	31.040	9.489	23.723	10.520
4	38.800	11.842	29.605	10.117
5	46.560	14.198	35.495	9.741

**Table 3:** The range of process parameters set in this study.

Factor	Description (Unit)	Minimum Value	Maximum Value
A	Melt temperature (°C)	20	35
B	Curing pressure (MPa)	30	60
D	Curing time (s)	5	10

**Table 4:** Experimental results of process parameters and optimization objectives based on the LSH method.

Items	Melt Temperature (°C)	Curing Pressure (MPa)	Curing Time (s)	Residual Stresses (MPa)	Clamping Force (ton)
1	33.954	47.015	8.994	7.974	11.282
2	20.197	48.245	8.260	9.149	11.566
3	25.540	37.188	7.475	9.448	8.959
4	23.448	32.392	9.628	9.088	7.873
5	30.419	59.795	5.045	8.887	14.271
6	26.883	42.566	5.822	9.604	10.248
7	32.813	53.071	6.752	8.488	12.710
8	21.555	41.799	9.336	8.972	10.085
9	27.818	55.994	6.163	8.945	13.407
10	31.276	34.249	7.697	9.040	8.289

**Table 5:** Comparison of Process Optimization Results for Manufacturing.

	Melt Temperature (°C)	Curing Pressure (MPa)	Curing Time (s)	Residual Stresses (MPa)	Clamping Force (ton)
**Pre-optimization**	30.419	59.795	5.045	8.887	14.271
**Post-optimization**	34.92	33.94	9.97	7.733	7.544
**Optimization rate (%)**	-	-	-	12.98	47.14

## Data Availability

The data presented in this study are available on request from the corresponding author.
